# Interactions Between Visual Working Memory, Attention, and Color Categories: A Pupillometry Study

**DOI:** 10.5334/joc.208

**Published:** 2022-02-07

**Authors:** Thomas Wilschut, Sebastiaan Mathôt

**Affiliations:** 1 University of Groningen, Faculty of Science and Engineering, Department of Behavioral and Cognitive Neurosciences, Netherlands; 2 University of Groningen, Faculty of Behavioral and Social Sciences, Department of Experimental Psychology, Netherlands

**Keywords:** Visual working memory, visual attention, pupillometry, color, categories

## Abstract

Recent studies have found that visual working memory (VWM) for color shows a *categorical bias*: observers typically remember colors as more prototypical to the category they belong to than they actually are. Here, we further examine color-category effects on VWM using pupillometry. Participants remembered a color for later reproduction on a color wheel. During the retention interval, a colored probe was presented, and we measured the pupil constriction in response to this probe, assuming that the strength of constriction reflects the visual saliency of the probe. We found that the pupil initially constricted most strongly for non-matching colors that were maximally different from the memorized color; this likely reflects a lack of visual adaptation for these colors, which renders them more salient than memory-matching colors (which were shown before). Strikingly, this effect reversed later in time, such that pupil constriction was more prolonged for memory-matching colors as compared to non-matching colors; this likely reflects that memory-matching colors capture attention more strongly, and perhaps for a longer time, than non-matching colors do. We found no effects of color categories on pupil constriction: after controlling for color distance, (non-matching) colors from the same category as the memory color did not result in a different pupil response as compared to colors from a different category; however, we did find that behavioral responses were biased by color categories. In summary, we found that pupil constriction to colored probes reflects both visual adaptation and VWM content, but, unlike behavioral measures, is not notably affected by color categories.

## Introduction

Interactions between visual working memory (VWM) and attention play a central role in perception (Bahle, Beck, & Hollingworth, 2018; Downing, 2000; Theeuwes, Kramer, & Irwin, 2011). On the one hand, attention determines which information is encoded (Schmidt, Vogel, Woodman, & Luck, 2002) and maintained (Awh, Jonides, & Reuter-Lorenz, 1998; Griffin & Nobre, 2003) in VWM. On the other hand, VWM serves as a search template that guides attention towards objects that match the content of VWM (Chelazzi, Duncan, Miller, & Desimone, 1998; Desimone & Duncan, 1995; Frătescu, Van Moorselaar, & Mathôt, 2019; Hollingworth, Matsukura, & Luck, 2013; Olivers, Meijer, & Theeuwes, 2006; Wolfe & Gray, 2007; Zhou, Lorist, & Mathôt, 2020).

Such interactions between VWM and attention have been extensively studied, often using color as the relevant feature (Allred & Flombaum, 2014). In doing so, most studies have so far focused on *continuous* representations, which represent exact feature values, such as the exact hue of a color. *Categorical* representations, which represent the general class to which a stimulus belongs without representing its exact features, have only recently become a topic of investigation. For example, Bae, Olkkonen, Allred, and Flombaum (2015) recently found that color estimations drawn from VWM are biased away from color category boundaries towards category prototypes. The aim of the present study is to further examine how VWM for color influences visual attention towards color, as well as the importance of color categories here-in, using a new pupillometric technique that was recently introduced by Olmos-Solis, van Loon, and Olivers (2018; see also Binda & Murray, 2015).

The most-often-used paradigm to study interactions between VWM and attention is a combined memory-search task (e.g., Hollingworth et al., 2013; Hollingworth & Beck, 2016; Olivers et al., 2006). For example, in a study by Zhou et al. (2020), participants remembered two colors for later reproduction on a color wheel. During the retention interval, a search display was shown. The participants identified a target, defined by having a unique shape, as quickly as possible. Zhou and colleagues found that participants responded faster on the search task if the target color matched one of the memory colors (as compared to targets having another color), whereas they responded slower if a distractor matched one of the memory colors (as compared to distractors having other colors). These results reflect *memory-driven guidance*: attention is guided towards memory-matching stimuli, thus facilitating search when the memory-matching stimulus is the target, and interfering with search if it is a distractor.

Research on VWM also often relies on *pupillometry*, the measurement of the size of the eye’s pupil. There are three main ways in which pupillometry has been used in VWM research, and each reflects a different aspect of VWM. First, early work initiated by Kahneman and Beatty (1966) demonstrated that pupil size reflects working memory load: the higher the mental effort or load on working memory (for example, the higher the number of items the subject needs to maintain), the more strongly the pupil dilates (Einhäuser, 2017; Kahneman & Beatty, 1966); this technique uses pupil size as a measure of a cognitive load. Second, Hustá, Dalmaijer, Belopolsky, and Mathôt (2019) and Zokaei, Board, Manohar, and Nobre (2019) showed that the pupillary light response (a constriction of the pupil in response to bright stimuli) is affected by VWM content: pupils were smaller when participants maintained bright stimuli, as compared to dark stimuli, in VWM; this technique uses cognitive modulations of the pupil light response as a window onto the content of VWM (Mathôt & Van der Stigchel, 2015). Third, the strength of pupil constriction in response to a task-irrelevant probe has been used to measure the extent to which the probe matches the content of VWM (Olmos-Solis et al., 2018); this approach, which we will use for the present study, and which will be explained in more detail below, is similar in its logic to the combined memory-search paradigm discussed above.

As a precursor to using pupil constriction as measure of interactions between attention and VWM, Binda and Murray (2015) used pupil constriction to measure the locus of covert visual attention. In their study, participants fixated at the center of the screen, while they covertly attended to either the left or the right side of the screen. Next, a task-irrelevant probe was presented. The probe was either presented at the attended side of the screen, or at the other side. Crucially, the authors found that the pupil constricted more when the probe was presented within, as compared to outside of, the focus of attention. In other words, Binda and Murray showed that the pupil constricts more strongly in response to attended (and therefore visually salient) probes than in response to unattended probes.

Olmos-Solis et al. (2018) built on the study by Binda and Murray (2015) to investigate interactions between attention and VWM. Olmos-Solis and colleagues used a working-memory task in which a color was shown to the participants, which they needed to remember in order to complete a later recognition task. During the retention interval, task-irrelevant colored probes were shown to the participants. Probes could either have the exact same color as the memory item, or a different color (randomly sampled from six evenly spaced alternative colors). Olmos-Solis and colleagues found that, while the initial pupil constriction did not differ between conditions, pupil size returned to baseline more slowly for color stimuli that matched the content of VWM than for stimuli that did not match the content. This prolonged pupil constriction for matching vs non-matching stimuli likely reflects that stimuli that match the content of VWM capture attention more strongly, and perhaps also for longer, than non-matching stimuli do.

There is a subtle-yet-crucial difference between the findings by Binda and Murray (2015) and Olmos- Solis et al. (2018): whereas Binda and Murray found that covert attention increases the *strength* of the initial pupil constriction in response to a stimulus, Olmos-Solis and colleagues found that memory-driven attentional capture *prolonged* the pupil constriction to memory-matching stimuli. These results imply that attention can modulate pupil constriction in at least two distinct ways, which possibly reflect different levels of processing at which attention operates on visual representations.

In the present study, we aim to replicate and extend the results of Olmos-Solis et al. (2018) by testing whether pupil constriction is enhanced not only for stimuli that exactly match VWM content, but also for stimuli that do not exactly match VWM content, but do share the same category. In other words, we test whether pupil constriction is prolonged, for example, for any shade of red while any other shade of red is maintained in VWM. In doing so, we build on recent studies showing that categorical biases are a crucial aspect of VWM. For example, as already briefly mentioned above, Bae and colleagues (2015) conducted two behavioral experiments to examine the effects of color categories on VWM. They used a delayed estimation task in which a color was shown to a participant. Participants were asked to remember and reproduce the color as accurately as possible after a short retention interval. In addition, Bae and colleagues asked a second group of participants to indicate prototypical colors of different color categories (e.g., the reddest shade of red), as well as the boundaries between two adjacent categories (e.g., a shade that sits perfectly in-between red and pink). They found that responses drawn from working memory were significantly biased away from category boundaries towards category prototypes. For example, yellowish shades of green were reproduced as a more prototypical green than they actually were. Based on their results, Bae and colleagues proposed a ‘dual-content’ model: they proposed that VWM for color relies both on continuous representations (exact hue values) and categorical representations.

While relatively new in VWM research, the notion that color perception is heavily influenced by categories has a rich history in linguistics and perception research (Gegenfurtner & Kiper, 2003). For example, most languages use only a small number of color terms, around 11, while people are able to distinguish up to 100,000 different color shades (Linhares, Pinto, & Nascimento, 2008; Pointer & Attridge, 1997). Another source of evidence for category effects on color perception comes from color-discrimination tasks: people are better able to distinguish between-category color pairs than within-category color pairs (Winawer et al., 2007; Witthoft et al., 2003). In other words, two different colors that belong to the same category are perceived as being more similar to each other than two colors that belong to different categories, even when the two color pairs are equally dissimilar in color space (Witzel & Gegenfurtner, 2014).

In this study, we measure pupil constriction in response to task-irrelevant colored probes that do, or do not, match the content of VWM. As a crucial extension to Olmos-Solis et al. (2018), we include not only matching and non-matching probes, but also ‘slightly mismatching’ probes that belong either to the same or to a different category as the color that is maintained in VWM. The aim of our experiment is threefold. First, we aim to replicate the finding that memory-matching stimuli elicit a more prolonged pupil constriction than non-matching stimuli do, thus validating this specific use of pupillometry as a valuable technique for VWM research. To foresee the results, we indeed replicated this finding, but only when contrasting matching stimuli with ‘slightly mismatching’ stimuli (relatively close to the memory color on a color circle). Strikingly, when contrasting matching stimuli with ‘completely mismatching’ stimuli (180° away on a color circle), we found a very different pattern, such that the initial pupil constriction was strongest for completely mismatching stimuli, possibly due to a lack of adaptation for these colors.

Second, after replicating the results of Olmos-Solis and colleagues, we aim to extend their findings by testing whether slightly mismatching stimuli cause a more prolonged pupil constriction when they share the same color category as the memory color (as if they were matching stimuli), as compared to when they do not. To foresee the results, we found no difference in the pupil response to slightly mismatching stimuli as a function of whether or not they share the same category as the memory color; this suggests that color-category effects in VWM do not notably affect the levels of visual processing that are reflected in our pupil-size measure.

Third, in order to examine color-category effects both from a pupillometric and a behavioral point of view, we aim to replicate the behavioral findings from Bae et al. (2015). To foresee the results, we replicated the finding that behavioral color reproductions are strongly biased towards category prototypes. This suggests that, although not reflected in pupil size, color categorization did occur in our task.

## Methods

Methods, hypotheses, and sampling and analyses plans for this study were preregistered on the Open Science Framework before the start of data collection (see: *https://osf.io/w56cm/*; hypotheses, and sampling and analyses plans listed under ‘Experiment 1’ refer to those described in the current paper). Deviations from the preregistration are indicated where applicable. All data, analysis scripts, and supplementary materials are available on *https://osf.io/qksfh/*.

### Participants and Procedure

Thirty first-year psychology students participated in the experiment. All participants had normal or corrected-to-normal vision and normal color perception. Participants received course credit for participation. All participants gave written informed consent and the study was approved by the ethics committee of the department of psychology at the University of Groningen (study approval code: PSY-2021-S-0321).

**Figure 1:**
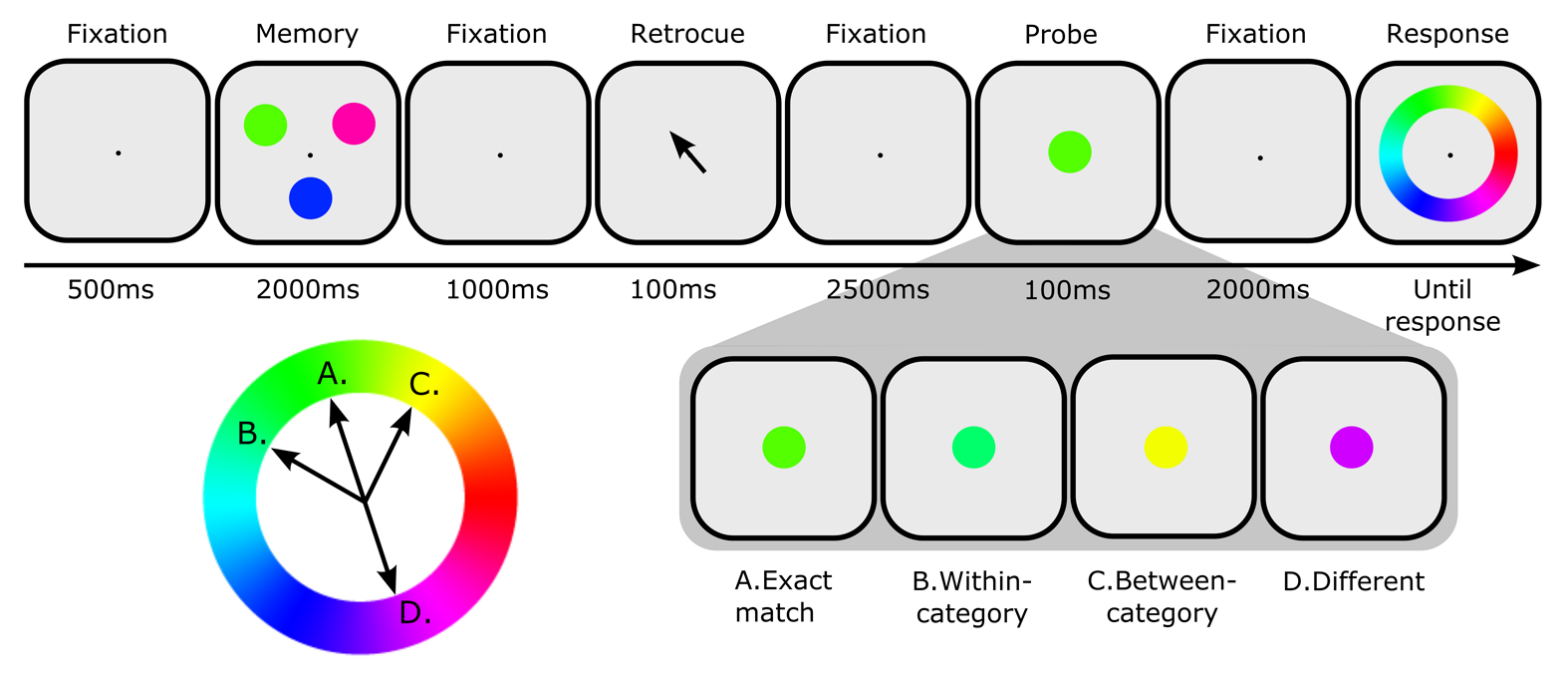
Trial sequence. Participants were asked to remember the color of a retro-cued circle. During the retention interval, one of four colored probes was shown. Next, the participant was instructed to indicate the memory color on a color wheel.

In brief, participants remembered three colored disks; after a short retention interval, one of the disks was indicated by retro-cue for later reproduction on a color wheel (see ***Figure 1***). On *Probe* trials, a task-irrelevant probe was presented after the retro-cue; for those trials, we analyzed pupil constriction in response to the probe (but not behavioral responses). On *No Probe* trials, no such probe was presented; for those trials, we analyzed the behavioral responses (but not pupil responses).

#### Probe trials

Each trial started automatically when the participant fixated at a cross at the center of the screen. Three colored circles (radius = 1.3° visual angle) were shown for 2000 ms. The colors were taken from a hue-saturation-value (HSV) color circle with full saturation and value. For two of the circles, the hue was selected fully randomly; for the remaining circle (the ‘memory color’ that would be retro-cued later) the hue was selected following the color-selection procedure described below. After a 1000 ms fixation screen, an arrow pointing to the to-be-remembered circle (the retro-cue) appeared for 500 ms. Following another 1000 ms fixation screen, a task-irrelevant probe was shown for 100ms. Participants were told that the probe was irrelevant for the task. The probe could be (A) an *exact match*: the exact same color as the memory color that the participant was maintaining in memory; (B) *within-category*: (slightly) different from the memory color, but in the same color category (in ***Figure 1***: green); (C) *between-category*: equally different from the memory color as probe (B) in HSV color space, but in another color category (in ***Figure 1***: yellow) or (D) *different*: completely different from the memory color. After another 2000 ms, participants were asked to reproduce the memory color by clicking on a color circle. Importantly, we were not interested in the behavioral responses on probe trials, because the probe color likely distorts the representation of the memory color. Rather, the memory task served to motivate the participants to remember the memory color. In probe trials, pupil size after probe presentation was the main outcome variable.

#### No Probe trials

Because the probe would likely interfere with the reproduction of the memory color, we also included trials on which no probe was presented, but which were otherwise identical to the Probe trials. In these trials, we closely followed the design adopted by Bae and colleagues (2015), in which participants were asked to remember and reproduce a color. In probe trials, the performance on the behavioral task (in which participants were asked to reproduce the memory color by clicking on a color circle) was the main outcome variable.

All participants completed 190 experimental trials, 152 Probe trials (38 trials for each Probe condition) and 38 No Probe trials. Trial order was fully randomized. The experiment was split into eight blocks of approximately equal length. Participants received feedback on the accuracy of their memory color reproductions using a point reward system (the more accurate the color estimations, the more points a participant could earn. High scores were saved and displayed to the participant after the completion of each block). Stimuli were presented using OpenSesame 3.3.5 (Mathôt, Schreij, & Theeuwes, 2012) and PyGaze toolbox (Dalmaijer, Mathôt, & Van der Stigchel, 2014). Stimuli were presented on a ProLite G2773HS monitor with a 1920 x 1080 pixel resolution and a 60 Hz refresh rate.

### Stimuli

Memory colors used in the experiment were randomly drawn from a hue-saturation-value (HSV) color circle with full value (i.e., maximum brightness without becoming white) and full saturation for each hue. Luminance ranged from 20.78 cd/m^2^ (yellow) to 9.89 to cd/m^2^ (blue). In a previous experiment conducted in our lab, 25 participants indicated boundaries between, and prototypes for, seven common color categories: green, blue, purple, pink, red, orange, and yellow (see: *https://osf.io/fprt9/*). The color-selection procedure for the selection of probes was based on these boundaries.

The bottom-left panel in ***Figure 1*** shows how the four different probe-type colors were sampled. Exact-match probes (A) always had the same hue as the memory color (the cued circle in the memory display). Within-category probes (B) were always slightly (at least 4° of hue angle) different from the Exact-match probes but belonged to the same color category. Between-category probes (C) were equally different from the Exact-match probe as the Within-category probe, but were sampled on the other side of the Exact-match probe on the color wheel, from another color category. In other words, Within- and Between category probes differed equally from the memory item continuously (measured in hue angle degrees) but belonged to a different color category. Crucially, Within- and Between-category hues were fully counterbalanced; that is, each Within-category hue also occurred once as a Between-category hue and vice versa. Different probes (D) were defined as the 180° opposite of the Memory hue. For more details on the color selection procedure, see *https://osf.io/w56cm/*.

The distributions of luminances for probes in the Between- and Within-category probes were identical by design (*M* = 15.03 cd/m^2^; *SD* = 2.59 cd/m^2^). Exact- and Different probe type luminance distributions were not identical but highly similar (*M* = 14.88 cd/m^2^; *SD* = 2.24 cd/m^2^; *M* = 15.48 cd/m^2^; *SD* = 2.25 cd/m^2^, respectively; see online Supplementary Materials, *https://osf.io/dgyr3/*).

### Pupillometry

Participants were seated in a dimly lit room, approximately 60 cm from the computer monitor, with their chin placed on a rest to reduce head movement. Pupil size and gaze position were recorded monocularly using the 1000 Hz video-based eye tracker EyeLink 1000 desktop mount (SR Research, Mississauga, ON, Canada). Before the start of the experiment, a nine-point eye-tracking calibration- and validation procedure was performed. Participants were instructed to keep fixating at the centre of the screen during the entire trial, and to keep blinking to a minimum.

## Results

### Pupil size: Preprocessing

Pupil size was only analyzed for Probe trials. Pupil time series were downsampled to 100 Hz and time- locked to the onset of the probe. Pupil size was recorded in arbitrary units and converted to millimeter (mm) of diameter; to determine the conversion formula, we measured the size of artificial pupils (black circles printed on white paper) of known sizes. Pupil size was baseline-corrected by subtracting the average pupil size during the first 50 ms after probe onset from each sample. Standardized average pupil sizes during the baseline period (0 – 50ms) after probe onset for each participant were computed (see: *https://osf.io/w56cm/*). Trials containing baseline pupil sizes larger than two standard deviations above (*z* ≥ 2) or below (*z* ≤ –2) the mean pupil size during baseline for the participant were considered outliers and excluded from the data. In total, 209 trials (3.37%) were excluded from the data based on this criterion. No data was excluded based on other criteria, such as recording quality or task performance; that is, no participants showed exceptionally noisy data, and all participants scored above chance on the memory test.

### Pupil size: Exact-match vs different probes

**Figure 2:**
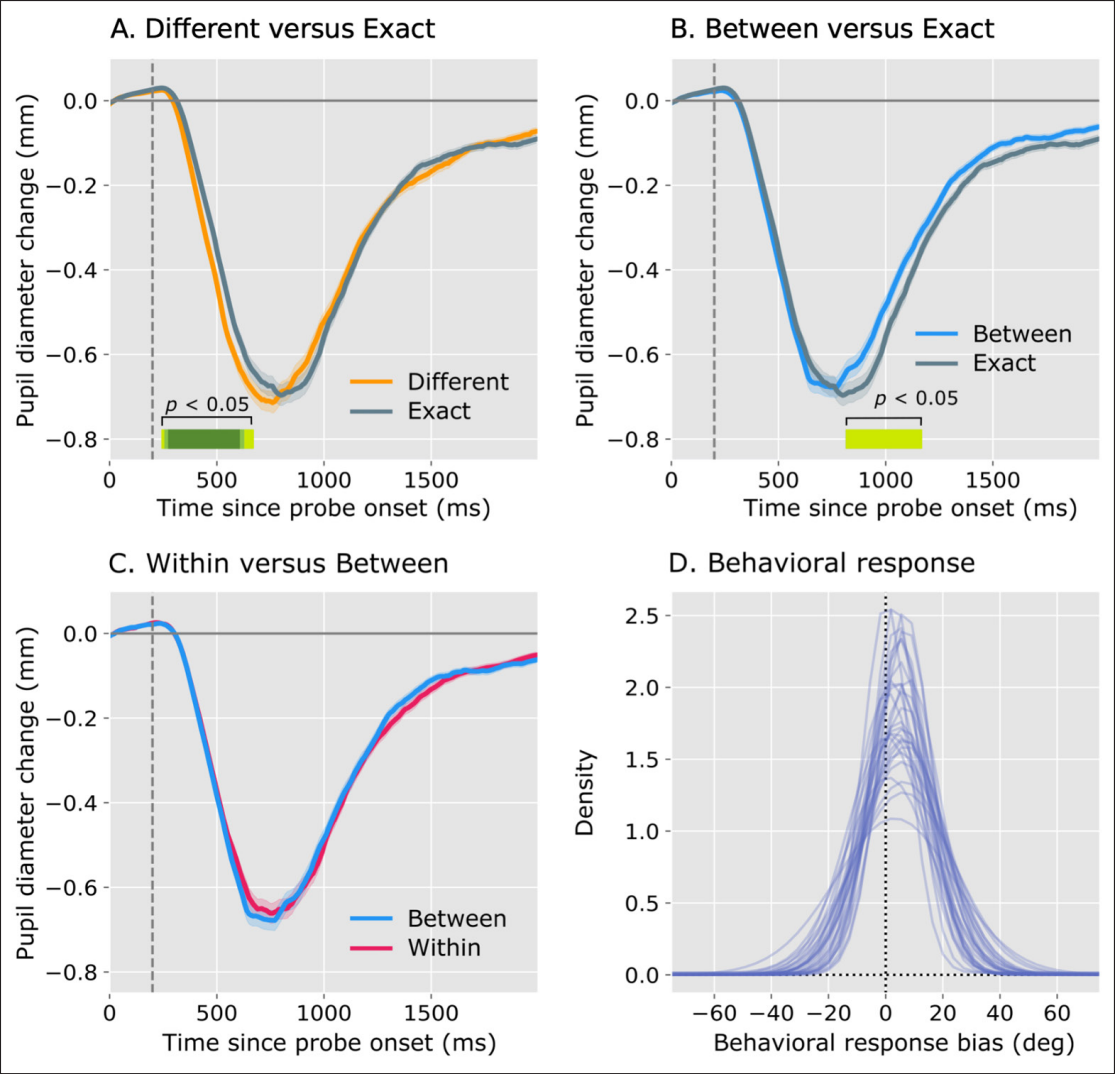
**(A-C)** Linear mixed effects model results for pupil response during probe presentation; **(D)** behavioral response bias. In A-C, the dotted line represents probe offset, and significant differences between pupil responses are indicated with the horizontal green line (yellow-green: *p* < 0.05; green: *p* < 0.01; dark green: *p* < 0.001). **(A)** Linear mixed effects model testing the difference in pupil response for exact-match versus different probes. Different probe types resulted in initial stronger pupil constriction compared to exact-match probe types. **(B)** Linear mixed effects model testing the difference in pupil response for exact-match and between-category probe types. Between-category probe types resulted in a slower pupil recovery compared to exact-match probe types. **(C)** Model testing the difference between between- and within-category responses. No significant differences between pupil responses for within- and between-category were found. **(D)** Behavioral response bias. Individual lines represent individual participant responses. Memory bias is shown on the x-axis (positive bias means an error towards category centers, negative bias means error away from category centers). In D, the dotted line represents unbiased, veridical color reproductions.

The first aim of this study was to replicate the results by Olmos-Solis et al. (2018). To do so, we initially focused on the contrast between Exact-Match and Different probes, see ***Figure 2A***. For each sample, we fitted a linear mixed effects model (lmer, using the R package lme4; Bates, Mächler, Bolker, & Walker, 2015) with Pupil Size as dependent measure, Probe Type (Exact Match vs Different) as fixed effect, and by-participant random intercepts and slopes. We considered an effect significant if *p* < 0.05 (as estimated by the R package lmerTest, (Kuznetsova, Brockhoff, & Christensen, 2017) for at least 20 samples, or 200ms, in a row.

Unlike we predicted, the initial pupil constriction was stronger in response to Different probes as compared to Exact-Match probes. Later in time, no significant differences were found anymore, suggesting a slower recovery for Exact-Match than for Different probes. These results paint a more complex picture than that suggested by Olmos-Solis et al. (2018). Specifically, it appears that our Different probes, which were maximally different from the memory color, were less affected by visual adaptation than the Exact-Match probes, and that this resulted in a more pronounced initial constriction; in turn, this may have obfuscated any later-emerging effects of memory-driven capture.

### Pupil size: Exact-match vs between-category probes

In the study by Olmos-Solis et al. (2018), the effect of memory-driven capture was investigated by contrasting Relevant probes (to follow their terminology; analogous to our Exact-Match probes) with either Irrelevant probes (which were presented but not cued) or Not Presented probes (which were not presented before). Crucially, however, the hues for the Irrelevant and Not Presented probes were not 180° opposite from the memory hue, and thus not comparable to our Different probes. Rather, these probes were somewhat similar to our Between-Category probes, in the sense that they were different from the memory color and also belonged to a different color category, but that they were usually not that far away from the memory color in HSV color space.

Therefore, we conducted an exploratory analysis in which we contrasted Exact-Match with Between- Category probes (see ***Figure 2B***), using a similar analysis as described above for the Exact-Match vs Different contrast. We now found that Exact-Match probes resulted in a more prolonged pupil constriction as compared to Between-Category probes (but no difference in the strength of the initial pupil constriction). Possibly, visual adaptation is fairly general and affected Exact-Match and Between-Category probes (which were not that far away from each other in HSV color space) to a similar degree, thus strongly reducing the effect on the initial constriction strength that we found above when contrasting Exact-Match and Different probes. As a result, we can now more clearly observe the effect of memory-driven capture per se, which, as reported by Olmos-Solis et al. (2018), indeed results in a prolonged pupil constriction.

### Pupil size: Within- vs between-category probes

The second aim of this study was to examine whether memory-driven capture, as measured through the strength of pupil constriction, is modulated by categorical status. To test this, we conducted an analysis in which we contrasted Within-Category with Between-Category probes (see ***Figure 2C***), using a similar analysis as described above for the Exact-Match vs Different contrast. We found no difference; that is, in contrast to our prediction, pupil constriction was not more prolonged in response to Within-Category probes as compared to Between category probes, suggesting that, at least at the level that pupil responses reflect, color-category boundaries do not affect memory-driven capture.

### Behavior: Categorical bias

The third and final aim of this study was to replicate the main findings by Bae et al. (2015), who showed that responses drawn from working memory are biased away from color-category boundaries towards category prototypes. For No Probe trials only, we analyzed performance on the memory task, using the same category ratings as described under *Stimuli*. (For completeness, we also analysed the behavioral responses for Probe trials. This analysis can be found in the online Supplementary Materials at *https://osf.io/dgyr3/*.) For each participant separately, we fitted a mixture model to the distribution of the response errors (towards category prototypes) in the memory search task (no colored-singleton trials only; see Zhou, Lorist, & Mathôt, 2021 for details of the biased-memory toolbox for model fitting). This resulted in three parameters: the guess rate ([0,1]) which reflects the proportion of responses resulting from random guessing; the precision ([0,10.000]), which reflects the narrowness (i.e. the inverse of the variance) of the response distribution; and the response bias ([–180,180]), which reflects the extent to which memory responses are biased towards category prototypes (i.e. reproducing a yellowish shade of green as a more prototypical green than it actually was).

***Figure 2D*** shows the estimated distributions (i.e. the model fits) of response errors towards color category prototypes. Individual lines correspond to individual participants. Overall, the guess rate ranged between 0–10 %, and the precision parameter ranged from 442–2880, which are values that, based on our experience, fall within the normal range for a working memory task with a set size of three. More importantly, we conducted a one-sample *t*-test to test if the response bias deviated from zero. Confirming our hypothesis, and replicating the results of Bae et al. (2015), we found a strong and positive color-category bias (*t(29)* = 11.152, *p* < 0.001). On average, responses were biased 4.53° (SD = 2.23°) away from category boundaries towards category prototypes. In other words, even though pupil constriction was not affected by category boundaries, behavioral responses were.

## Discussion

In this study, we used pupillometry to investigate attentional capture by stimuli that match the content of visual working memory (VWM) for color, and how such memory-driven capture is affected by categorical biases. We extended the paradigm introduced by Olmos-Solis et al. (2018), who found that task-irrelevant probes that match a color that is maintained in VWM trigger a more prolonged pupil constriction than probes that do not match the color maintained in VWM; this prolonged constriction likely reflects that memory-matching stimuli capture attention and are therefore perceived as more salient than non-memory-matching stimuli (see also Olivers et al., 2006). A crucial difference between our study and previous work is that we included different classes of non-matching probes: probes could be maximally different (180° apart on a HSV color circle) from the memory color; probes could be slightly different from, and from a different color category than, the memory color; or probes could be slightly different from, and from the same color category as, the memory color. This subtle manipulation has yielded several key insights, some of which we had not anticipated and should be confirmed in future studies on the relationship between pupil size, attention, and VWM.

Overall, our results replicate the findings of Olmos-Solis and colleagues but with an important qualification: Olmos-Solis and colleagues randomly selected non-matching probes from six color categories. In our study, we used three different classes of non-matching probes, and we therefore asked which of our non-matching probes was most comparable to those used by Olmos-Solis and colleagues. We focused on “between-category” probes, which were always from a different color category than the memory color, but on average still relatively close to the memory color, just like the non-matching probes used by Olmos-Solis and colleagues—and we indeed found that memory-matching probes triggered a more prolonged pupil constriction than non-matching (between-category) probes did.

In line with many behavioral studies (Hollingworth et al., 2013; Hollingworth & Beck, 2016; Olivers et al., 2006; Zhou et al., 2020), this prolonged pupil constriction likely reflects memory-driven capture, which enhances the visual saliency of memory-matching stimuli. Crucially, however, the effect of memory-driven capture on pupil size is qualitatively different from the effect of covert visual attention on pupil size. More specifically, when a probe is presented at an attended as compared to an unattended location, the *strength* of the initial pupil constriction is affected (Binda & Murray, 2015), whereas memory-driven capture mostly affects the *duration* of the constriction (or, phrased differently, the speed of recovery to baseline). In other words, our pupil-size results suggest that memory-driven capture intervenes at a later stage than covert visual attention does. One interpretation of these findings is that, at least in the current paradigm with single, centrally presented probes, memory-driven capture mostly enhances attentional dwell time, such that attention lingers at memory-matching probes for longer (Parks & Hopfinger, 2008).

We also unexpectedly found that the initial pupil constriction was strongest for probes that were maximally different (180° away on a HSV color circle) from the memory color; this suggests that, early in time, this specific class of non-matching probes is actually *more* salient than memory-matching probes. A parsimonious explanation for this effect is one in terms of visual adaptation: the more a color resembles a color that has been presented before, the more adaptation occurs, and the lower its visual salience (McDermott, Malkoc, Mulligan, & Webster, 2010). On average, visual adaptation may have affected all of our probe types (which were all relatively similar to the memory color) about equally, except for the maximally different probes, for which relatively little adaptation occurred. In terms of pupil constriction, it appears that lack of adaptation enhances visual saliency in a similar way as covert visual attention does (Binda & Murray, 2015). This finding also suggests leads to pragmatic advice for future studies on the interaction between attention and VWM: when contrasting memory-matching with non-matching stimuli, the ‘purest’ non-matching stimuli are not too different from the memory stimuli, but rather (as a rough guideline) taken from adjacent categories.

Another aim of our study was to examine whether memory-driven capture, as measured through pupil responses, is affected by category boundaries; specifically, we predicted that non-matching “within- category” probes, which shared the same color category as the memory color, would lead to a prolonged constriction (as if they were matching probes) as compared to non-matching “between category” probes, which were sampled from a different category. However, there was no difference in the pupil response to within- vs between-category probes. Importantly, we did find that behavioral responses were affected by color categories, such that participants reproduced colors as more prototypical than they really were (Bae et al., 2015).

The above findings lead to the question: If VWM is affected by color categories when measured through behavior in a delayed-reproduction task, then why isn’t it similarly affected by color categories when measured through pupil responses? Bae et al. (2015) proposed a dual-content model in which VWM representations consist of two ‘channels’: a continuous channel, which (in the case of color) represents the exact hue; and a coarse channel, which only represents the category. Behavioral responses appear to be driven by a mix of these two channels, in the sense that responses in a delayed-reproduction task are slightly biased by, but not fully determined by, color categories. A recent study by Ester, Sprague, and Serences (2020) used decoding of electroencephalography (EEG) to show that, already less than 300 ms after stimulus onset, the neural response to a stimulus that is not prototypical for its category is biased so that it resembles the neural response that would have been elicited by the category prototype; phrased differently, EEG signals shows similar categorical biases as behavioral responses, suggesting that these categorical biases emerge very rapidly. With respect to the current results, this seems to rule out the possibility that pupil constriction isn’t affected by categorical biases because such biases emerge too slowly. Rather, it may be that pupil constriction as measured in the current paradigm (i.e. a reflexive light response to a task-irrelevant probe) largely reflects a low level of visual processing that is not, or hardly, affected by color categories. In addition, our pupillary and behavioral measures cannot be seen as different measures of the exact same concept: Behavioral bias reflects a tendency to remember a color as prototypical for its category, whereas a categorical effect on pupil size would measure if colors that belong to the same category are visually more similar as compared to colors from another category. Future research is needed to determine exactly what kind of neural activity is reflected in pupil constriction (which is still largely unclear), and to what extent categorical biases in perception in VWM are already part of the initial feed-forward sweep of visual processing, or whether they emerge as a result from top-down signals related to language and long-term memory.

In summary, we found that pupil responses show (1) a stronger initial pupil constriction in response to new stimuli that the participant has not previously seen (adaptation), and (2) a more prolonged pupil constriction in response to stimuli that match the content of VWM (memory-driven capture). We propose that the visual saliency of a stimulus is affected by both adaptation and VWM, and that these affect early and late components of the pupil response, respectively. Furthermore, (3) color categories do not affect memory-driven capture as measured through pupil constriction, even though (4) behavioral responses are biased by color categories in the same task. We propose that, while categorical biases are an important characteristic of visual perception and VWM, they may not affect all levels of visual processing. These results are important in two ways. First, by replicating and extending the results by Olmos-Solis and colleagues (2018) we show that this specific use of pupillometry is a valuable technique for VWM research. Second, these results contribute to our understanding of the mechanisms underlying categorization in VWM by suggesting that categorical biases may not affect all levels of visual processing equally.

## Data Accessibility Statement

All data, analysis scripts, and supplementary materials for this study are available on *https://osf.io/qksf9h/*.
